# AN AGENDA FOR THE FAMILY CARE GAP: ALIGNING SCIENCE, FAMILY EXPERIENCES, AND PUBLIC HEALTH

**DOI:** 10.1093/geroni/igad104.0057

**Published:** 2023-12-21

**Authors:** Joseph Gaugler

**Affiliations:** University of Minnesota, Minneapolis, Minnesota, United States

## Abstract

When considering the public health ramifications of family caregiving, a major concern is the future supply of family, friends, or other unpaid caregivers (hereafter referred to as family caregivers) of older persons with health needs. This presentation will summarize the state of the science on estimating the future “supply” of family caregivers for older persons, and whether there are structural or social determinants, in addition to long-standing socio demographic trends, which may contribute to the family care gap. In addition, the presentation will outline a potential research agenda on the family care gap that summarizes the potential implications of this phenomenon as well as how it aligns with efforts to elevate family caregiving for older people (particularly those living with dementia) as a matter of public health import.

